# Regulating the Nano–Bio Interface: Converging Electrochemical and Photonic Biosensing for Wearable Diagnostics

**DOI:** 10.1021/acs.nanolett.6c00860

**Published:** 2026-06-17

**Authors:** Vivek Kamat, Kaixuan Xu, Huijin An, Yayun Du, Sharon M. Weiss, Shekhar Bhansali

**Affiliations:** Department of Electrical and Computer Engineering, 5718 Vanderbilt University, Nashville, Tennessee 37240, United States

**Keywords:** wearable sensors, biosensors, nanomaterials, diagnostics, interface

## Abstract

The development of wearable biosensors has accelerated due to the combination of nanomaterials, integrative electronics, and miniaturized transduction mechanisms, enabling continuous monitoring of physiological and environmental markers. Electrochemical and photonic modalities have been shown to exhibit complementary capabilities in wearable applications, with each offering distinct advantages in sensitivity, selectivity, miniaturization, and power efficiency. Beyond single-modality functionality, the next generation of wearable diagnostics integrates electrochemical and optical transduction within the same platforms. In this short review, we examine the physical and functional demands of such convergence in wearable systems, highlighting model electrochemical systems, such as textile-integrated wound monitoring and hormone sensors, and how optical modalities provide orthogonal observables of interfacial and biochemical conditions. We further explore new emerging device architectures that leverage electrochemical and optical interrogation to generate robust, information-rich data sets, supporting long-term, real-world deployment of wearable diagnostic technologies for personalized healthcare and continuous monitoring applications.

## Graphical abstract

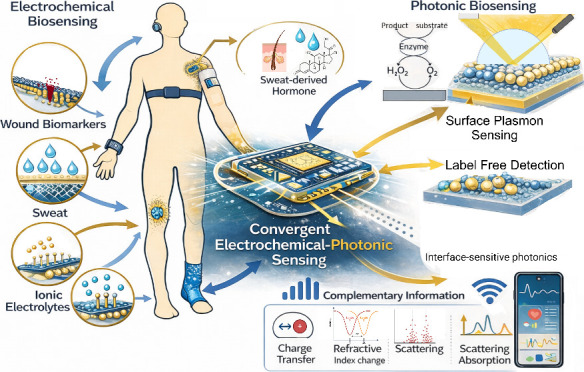

Wearable diagnostics has become an excellent category of sensing technologies that can interface with the human body and its immediate environment, providing continuous real-time sensing.^1^ The most common types of biosensors used as wearable devices have been optical and electrochemical biosensors, as these devices are compatible with miniaturized electronics and low power usage, and can directly transduce optical and biochemical reactions into electrical signals.^2^ Such properties have allowed a wide range of wearable platforms, such as smart dressings to monitor wounds,^3^ sweat- and saliva-based hormone detection,^4^ and transcutaneous gadgets to monitor volatile biomarkers. Specifically in the case of photonic biosensing,^5^ which includes optical absorption, scattering, and fluorescence, plasmonic, and the surface-enhanced spectroscopies, it has evolved at an accelerated rate and offers label-free and high sensitivity to interfacial phenomena,^6^ as well as noncontact interrogation of soft and flexible substrates. Although electrochemical and photonic biosensors excel at different aspects of the sensing interface, they address related yet distinct elements. Electrochemical transduction by its very nature is sensitive to charge transfer, ion conduction, and redox processes at the electrode/electrolyte interface, whereas photonic transduction is responsive to optical property changes due to the adsorption of molecules on the electrode/electrolyte interface, electrode/electrolyte interface morphology, and local dielectric environment.^7–10^

At dynamic nano–bio interfaces, electrical and optical responses are inherently linked, reflecting different aspects of a single, continuously evolving interfacial state (Figure 1). This extensiveness is driving a paradigm shift in perceiving electrochemical and photonic sensors as opposing modalities, toward their integration into single wearable systems.^11^ New wearable electrochemical platforms offer a solid base of such convergence.^12–14^ Textile-based wound monitoring systems are a demonstration where electrochemical sensors are imprinted in soft deformable matrices to provide continuous biochemical monitoring at the skin–device interface.^15,16^ Selective multiplexed recognition layers with nanostructured electrodes detect specific biomarkers, which are difficult to detect using conventional technology, has been demonstrated by wearable cortisol sensors and other hormone-detecting sensors.^17,18^ Simultaneously, optical biosensing modalities are complementary to these electrochemical methods; they can directly measure non-electrical properties, e.g., molecular interactions, surface kinetics, and interfacial architecture, in many cases without electrical contact or perturbation of the sensing surface.^19^

**Figure 1:**
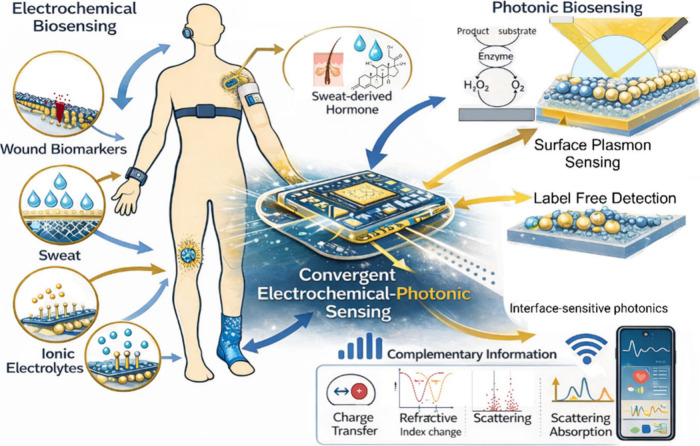
Electrochemical and photonic modalities jointly interrogate shared nano–bio interfaces, enabling complementary charge-based and optical readouts for robust, information-rich wearable diagnostics.

Integrated electro-optical microsystems, such as lab-on-a-chip systems that combine electrical transduction with optical detection to analyze rare cells, are evidence of convergent architectures in implementation.^20–22^ These integrated systems form a design intervention in which electrical and optical signals are co-acquired and co-interpolated to increase sensing fidelity and data harvesting. We believe that the merging of electrochemical and photonic biosensing is a natural and inevitable development for wearable diagnostics, as highlighted in this review. Instead of ruling convergence as just multimodal redundancy, we present it as a systems-level approach to the concerted design of electrochemical and optical modalities that can probe the nano–bio interface in complementary physical senses. Our vision is that these convergent biosensing systems will simplify and strengthen wearable diagnostic systems, providing more informative, versatile, and scalable solutions. Recent reviews have extensively summarized electrochemical biosensors, optical biosensing systems, plasmonic wearable platforms, flexible photonics, and multimodal sensing architectures individually. Most of the previous reviews, however, regard both electrochemical and photonic modes as parallel sensing modalities and not as coupled observations of a dynamic and evolving nano–bio interface (Table 1). This distinction becomes more significant for long-term wearable applications, where the sensitivity and selectivity of the sensor are affected not only by the sensitivity and selectivity of the sensing material but also by biofouling, the hydration-dependent transport of the sensing material, the restructuring of the interfaces between the sensor and the environment, mechanical deformation, signal shift, and environmental perturbations. This Mini-Review is different from previous reviews, which were mostly devoted to material systems or analyte detection or to the integration of the device, in that it highlights the convergence of electrochemical and photonic transduction at nano–bio interfaces and how multimodal colocalized sensing can enhance interpretability, robustness, calibration fidelity, and long-term clinical viability of wearable diagnostics.

**Table 1:** Comparative Analysis of Recent Wearable Biosensing Reviews in Relation to the Present Mini-Review, Underlining Prevailing Sensing Paradigms, the Lack of Translation Issues, and the Mechanistic Electrochemical–Photonic Convergence Framework Introduced in This Work

Wearable biosensing domain	Representative recent review	Core sensing mechanism addressed	Unresolved limitations and gaps	Advances in this review
**Electrochemical wearable biosensing**	Kannan, P., & Maduraiveeran, G. (2026)^23^	Nanomaterial-enabled electrochemical microbiosensors, flexible electrodes, enzyme-mimetic catalysts, bioaffinity recognition, POC diagnostics	Strongly focused on electrochemical biosensors, sensitivity, selectivity, stability, and wearable POC formats; limited treatment of photonic observables and electrochemical–photonic colocalization	Reframes electrochemical readouts as charge-transfer, ion-transport, and impedance observables that can be paired with photonic signals from the same nano–bio interface
**Electrochemical wearable biosensing**	Malode, S. J., Alodhayb, A. N., & Shetti, N. P. (2026)^24^	Amperometric, potentiometric, and impedimetric sensing; nanostructured electrodes; aptamers; signal amplification	Discusses stability, multiplexing, biofouling, and low-power signal amplification, but remains primarily electrochemical	Extends beyond electrochemical signal optimization by positioning photonic readouts as orthogonal probes for drift correction, fouling interpretation, and interfacial validation
**General wearable biosensing**	Xian, X. (2026)^25^	Broad wearable health monitoring, sweat/tear/breath sensing, cardiovascular devices, wearable antennas, AI/wireless systems	Broad overview; highlights miniaturization, accuracy, precision, and real-world validation, but lacks mechanistic electrochemical–photonic interface analysis	Narrows the scope from broad wearable monitoring to the mechanistic regulation of shared nano–bio interfaces under real-world deformation, fouling, and drift
**Photonic/optical biosensing**	Tabassum, M. M., Vasimalla, Y., Singh, R., & Kumar, S. (2026)^26^	SPR, SERS, fluorescence, interferometric sensing, nanophotonics, microfluidics, AI/IoMT-enabled optical diagnostics	Strong optical focus; discusses optical signal attenuation, scattering, fabrication cost, and AI-guided calibration, but does not couple optical readouts with electrochemical charge-transfer processes	Positions optical signals as complementary observables of refractive index, adsorption, scattering, morphology, and dielectric changes occurring at the same electrochemical sensing interface
**Recognition-interface biosensing**	Xing, R., Chai, H., Liu, B., Wang, R., & Hu, S. (2025)^27^	Epitope-imprinted polymers, artificial antibodies, peptide-template recognition, disease diagnosis and therapy	Strong receptor-recognition focus; limited discussion of wearable integration, multimodal transduction, and long-term interface drift	Incorporates MIPs/aptamers as interfacial recognition layers within a larger electrochemical–photonic wearable framework governed by transport, fouling, and mechanical deformation
**Disease-specific nanomaterial biosensing**	Natarajan, B., Zhou, Q., Maduraiveeran, G., & Kannan, P. (2026)^28^	Metal nanocomposites, coordination chemistry, Aβ peptide detection, electrochemical POC diagnostics	Strong disease- and biomarker-specific analysis; focuses on Aβ detection, metal nanocomposites, stability, and POC workflow integration	Generalizes nanostructured interface engineering beyond one disease biomarker toward multimodal wearable platforms that use electrochemical and photonic channels for broader diagnostic robustness

Mechanistically, the electrochemical and photonic wearable biosensors are based on two related effects arising from the same nano–bio interfacial evolution. Electrochemical readouts are based on changes in electron-transfer kinetics and ion accumulation, double-layer structure, and faradaic accessibility, as well as changes in interfacial impedance, while photonic readouts rely on refractive-index modulation, optical scattering, plasmonic field enhancement, molecular adsorption, and changes in surface morphology. However, in realistic wearable environments, these processes are not independent; biofouling and hydration influence both charge-based and optical observables, as do mechanical strain, receptor reorganization, and analyte transport. Convergent electrochemical–photonic sensing can therefore offer a mechanistic approach to discriminate between the true biochemical events and artifacts arising from the deformation, fouling, optical attenuation, or electrochemical drift.

## 1 Wearable Biosensing as a Multiphysics Interface Issue

Wearable biosensors operate at complex nano–bio interfaces, where electronic, chemical, mechanical, and optical phenomena are reduced to nanometer dimensions and continuously influenced by physiological processes.^29^ The biochemical interactions are encoded in electrical and optical signals that react to surface chemistry, hydration, biofouling, changes in refractive index, absorption, scattering, and local dielectric environments. These reactions are dynamic in nature, and their properties are continually altered by the continual transport of molecules, cellular activity, mechanical deformation, and exposure to the environment. Therefore, wearable systems under dynamic conditions must function reliably at soft, wet, chemically active interfaces formed between nanomaterials and biofluids. Wearable diagnostics are therefore not to be considered as a one-dimensional sensing channel but rather as a multiphysics system in which the distinct transduction modalities provide complementary projections of a common interfacial state space. Awareness of this connection provides a robust foundation for integrating electrochemical and photonic biosensing models, enabling multimodal readouts to improve interpretability, robustness, and durability under physiological conditions.

## 2 Wearable Electrochemical Biosensing

### (a) Smart Dressing and Textile-Based Wound Monitoring

Electrochemical wound monitoring represents one of the most clinically actionable applications in wearable biosensing because of the highly rich biochemical dynamics that can directly indicate tissue damage, inflammation, and healing kinetics.^3,30–32^ The altered purine metabolism, oxidative stress, variable pH, and increased concentrations of inflammatory metabolites in acute and chronic wounds can be interrogated via electrochemical sensors in real time. The electrochemical sensors target xanthine, an inflammatory biomarker, and purine, in the purine catabolic pathway, which is produced when cells are injured and/or undergoing apoptosis.^33,34^ The xanthine concentration was used to predict wound severity and healing activities. The sensing approach follows a measurable electrical signal by using bienzymatic electrochemical architectures consisting of xanthine oxidase (XO) and horseradish peroxidase (HRP) in an enzymatic amplification of electron transfer (Figure 2).^18,35^ Multiwalled carbon nanotubes (MWCNTs) functionalized with gold nanoparticles (AuNPs) served as an active transducer surface to increase the electroactive surface area, efficiency in transporting the electrons, and loading of enzymes. This specific interface arrangement allowed for sensitivities on the order of 150 nA/uM of xanthine concentrations between healthy, inflamed, and highly injured tissues. Importantly, the wound sensors were tested on clinical samples obtained from wound dressings and perilesional tissues. These findings indicate the need to design interface-sensitive sensors with spatial location, nanostructured morphology, and biochemical selectivity as the three parameters that are co-optimized to trade off sensitivity with lifetime. However, enzyme instability, differences in wound fluid composition, protein fouling, peroxide-mediated degradation, pH changes in the wound, and mechanical delamination of the nanocomposite electrodes during extended wear-over periods are limitations of wound fluid sensing. The problem of wound monitoring is a typical example of the need for sensitivity alone being too simple to meet the need and where interface stability, antifouling design, spatially resolved sampling, and multimodal validation are key requirements.

**Figure 2:**
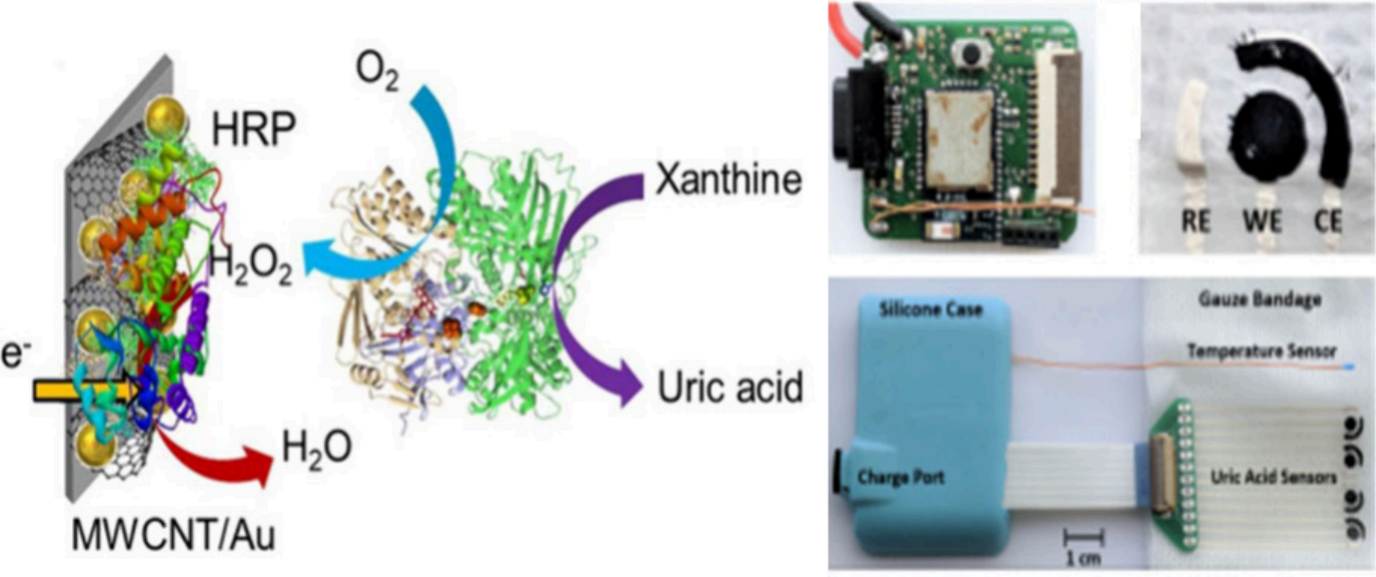
(a) Schematic illustration of the enzymatic electron-transfer mechanism during xanthine oxidation mediated by xanthine oxidase (XO) and horseradish peroxidase (HRP) on a (b) nanocomposite-functionalized electrode (electronics), (c) integrated with the wearable wound monitor.

### (b) Sweat- and Saliva-Based Electrochemical Hormone Monitoring

Wearable sensors are rapidly transforming noninvasive hormone monitoring by enabling continuous assessment of low-abundance biomarkers such as cortisol in sweat and saliva. Cortisol, the primary glucocorticoid stress hormone, exhibits a pronounced circadian rhythm and dynamic responses to acute and chronic stress, and while serum remains the reference matrix, sweat and saliva provide more accessible surrogates that correlate well with systemic levels in controlled studies.^36^ Recent work has delivered fully integrated on-skin patches and oral platforms capable of real-time or near-real-time cortisol readout in humans, positioning these devices for stress, mental health, and metabolic monitoring without repeated blood sampling (Figure 3).^37^ Central to this progress are nanostructured electrode architectures and advanced selective recognition layers that together address the intrinsically low cortisol concentration and complex backgrounds of sweat and saliva. Nanostructured carbons (laser-induced graphene, CNTs), conductive polymers, and metal or silica nanostructures increase the electroactive surface area, enhance charge transfer, and provide compliant interfaces that can be patterned on flexible or oral substrates.^37,38^ For example, mesoporous silica nanochannel-modified ITO combined with cortisol aptamers has been used in sweat-wearable formats with microfluidic sweat collection, achieving performance comparable to ELISA while operating directly on the skin.^39^ Similarly, high-surface-area graphene and nanofiber-based PEDOT/PAN scaffolds support ultralow detection limits in flexible aptamer sensors and floss- or mouthguard-like salivary platforms, maintaining sensitivity under repeated bending and real-sample conditions.^40,41^

**Figure 3:**
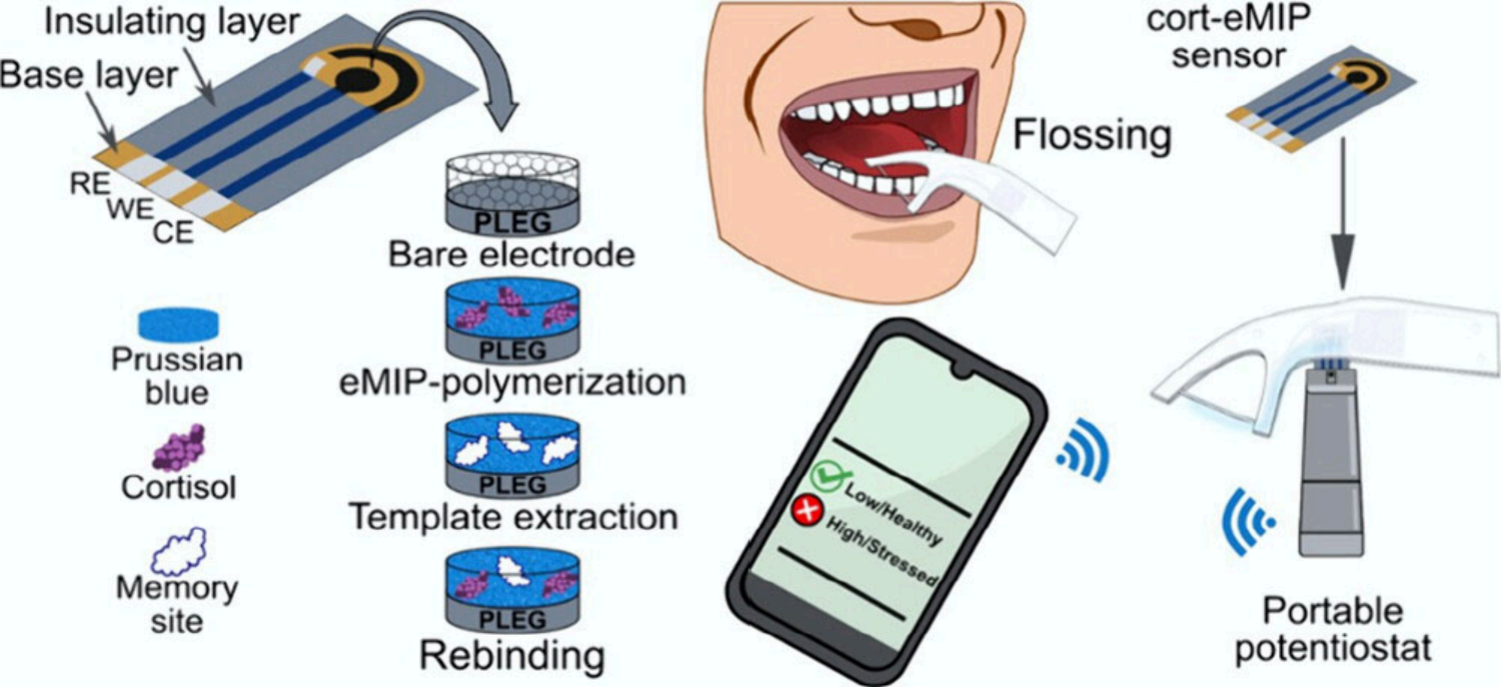
A sensing dental floss interfacing with a thread-based microfluidic channel and cortisol eMIP sensor fabricated using a porous laser-engraved graphene electrode (PLEG). Incorporating a polypyrrole-embedded redox-molecule (PPy/PB) with cortisol MIPs establishes a highly sensitive cortisol-sensing platform. Adapted and reprinted with permission (reference: 10.1021/acsami.5c02988).

Selective recognition layers are equally critical, as they must distinguish cortisol from structurally similar steroids, proteins, and other interferents present at higher levels in biofluids. Antibody-based competitive immunosensors remain widely used, particularly in multiplexed configurations such as the Stressomic sweat platform, which combines iontophoretic sweat induction, skin-interfaced microfluidics, and protein A/G-conjugated antibodies on nanostructured Au electrodes to simultaneously quantify cortisol, epinephrine, and norepinephrine with ELISA-comparable limits of detection.^42,43^ Aptamer-based systems, including conformation-switching aptamers on graphene or nanofiber electrodes, offer improved stability and the possibility of reagent-free or minimally labeled detection, while molecularly imprinted polymers (MIPs) provide fully synthetic, robust receptors compatible with harsh sweat environments and repeated use.^40,44,45^ In both sweat and saliva devices, these recognition layers are increasingly co-engineered with nanostructured electrodes and microfluidic modules (e.g., prestored reagents, sequential sampling, local pH control) to stabilize binding, mitigate matrix effects, and push detection limits into the low-nanomolar to picomolar range required for physiological profiling. However, key limitations persist, including signal drift and degradation of biological receptors under variable temperature and hydration. Matrix effects from pH, ionic strength, and skin/salivary contaminants limited longevity and stability over days. In addition to the low concentration of analytes, the major challenges for sweat- and saliva-based hormone sensing include variable secretion rates, biofluid dilution, diurnal fluctuations, nonspecific adsorption of steroids, receptor aging, and inter-user calibration transfer. Synthetic receptors, microfluidic sampling control, internal reference channels, and data-driven normalization strategies help to address these challenges. Future directions include replacing or reinforcing fragile biorecognition elements with robust synthetic receptors, such as engineered aptamers, proteins, MIPs, and hybrid polymer–biomolecule architectures integrated with microfluidics, to improve binding. With efforts to standardize performance metrics, fouling resistance strategies, and regulatory pathways, continuous hormone wearable sensors will move from proof-of-concept to clinical and consumer health tools.

### (c) Molecularly Imprinted Polymer-Based Hormone Detection

In addition to stress hormones, electrochemical wearable platforms have also been expanded to detect anabolic steroid hormones such as cortisol, estrogen, and testosterone, whose abnormal regulation has been associated with metabolic diseases, endocrine disorders, and aging.^46–48^ The most recent studies have shown molecularly imprinted polymer (MIP)-based electrochemical sensors that have the ability to detect testosterone without the use of labels through the creation of synthetic binding cavities that recapitulate the size, shape, and orientation of the functional groups of the steroid.^4^ The MIP-modified electrodes have redox transduction stability via interfacial change in charge-transfer resistance and accessibility of the Faradaic probe in the case of hormone binding, thereby allowing preference in quantifying testosterone in complex biofluids like serum and saliva. Notably, robust polymeric recognition layers avoid most of the disadvantages of antibody degradation and thermal instability and make testosterone sensing especially suitable for long-term, wearable operation. The incorporation of estradiol, testosterone, and cortisol into electrochemical wearable designs can be seen as part of a general trend toward multiplexed endocrine monitoring, in which the difference in hormone signatures can be resolved by engineering nano–bio interfaces, as opposed to using enzyme-specific reactions. Despite the thermal and chemical stability properties that they offer over antibodies, MIPs remain currently limited in their use to wearable applications due to the incomplete removal of the template, a heterogeneous distribution of binding sites, nonspecific adsorption, slow rebinding rates in complex biofluids, and lack of batch-to-batch reproducibility. Thus, the limit of detection is not the only parameter to be considered when assessing the future potential of MIP-based wearable sensors but also the imprinting factor, the possibility of receptor regeneration, fouling resistance, and operation duration upon repeated mechanical deformation.

## 3 Wearable Photonic Biosensing

Wearable photonic biosensors represent a key technology in noninvasive diagnostics, providing real-time diagnostic and molecular information based on high sensitivity and label-free detection capabilities. Wearable photonic biosensors can be organized into two practical classes: bulk tissue optics and interface-sensitive photonics, which include plasmonic or SERS-enabled molecular readouts and guided wave sensors. Bulk tissue optics underpins widely deployed modalities such as photoplethysmogram (PPG) and functional near-infrared spectroscopy (fNIRS), which estimate physiology from wavelength-dependent attenuation and scattering through vascularized tissue. This approach enables continuous tracking of pulsatile blood volume, oxygenation, and tissue hemodynamics in everyday form factors (Figure 4).^49^ Its limitations are equally clear: the measured signals are easily perturbed by motion, contact pressure, ambient light leakage, and interindividual differences in tissue optical properties and vascular dynamics. As a result, clinical-grade performance often depends on tight hardware–algorithm co-design, careful calibration, and standardized protocols that remain uneven across devices and use scenarios (Figure 5).^50–52^ In contrast, interface-sensitive photonics, exemplified by surface plasmon resonance (SPR) and related resonant nanostructures, as well as guided wave structures including fibers and integrated photonics, directly transduce nano–bio interfacial phenomena, molecular adsorption, binding kinetics, and local dielectric variations, offering a mechanistically interpretable complement to electrochemical sensing that is less dominated by bulk transport and matrix effects.^53^ While traditional SPR sensors that identify specific molecular fingerprints typically utilize benchtop measurements that are difficult to miniaturize, recent advances in localized SPR (LSPR) and surface-enhanced Raman scattering (SERS) have facilitated the integration of plasmonic structures onto flexible and stretchable substrates, such as PDMS, hydrogel, and functionalized textiles [c],^54^ [d, e].^55,51^ As one example, a flexible, stretchable, and single-molecule-sensitive SERS-active sensor was recently demonstrated, utilizing a heart-shaped gold nanodimer structure on a PDMS substrate to preserve optical performance under mechanical deformation, thereby extending interface-sensitive photonics toward wearable implementations [b].^55^ In general, by regulating the nano–bio interface through the deposition of a specific nanostructured material such as gold [f, g] ^52,56^ or silver [d, h] ^55,57^ onto flexible templates, SERS-based wearable sensors can achieve real-time multiplexed detection of biomarkers such as metabolites and pH in sweat or interstitial fluids [c, i, j],^54,58,59^ thereby bridging the gap between discrete laboratory assays and continuous wearable monitoring [k, l].^60,61^ Despite their advantages, the quantitative reliability of on-body SERS-wearable sensors is still limited by substrate reproducibility, biofluid variability, and mechanical and chemical stability under bending, sweating, and long wear.^62^

**Figure 4:**
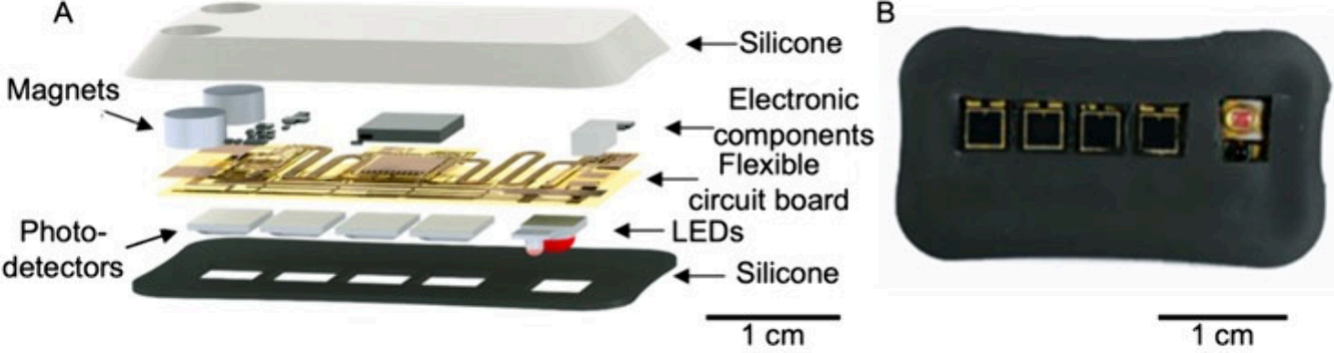
Representative multilayer architecture of a wearable fNIRS photonic sensing platform integrating emitters, photodetectors, flexible circuitry, and soft encapsulation for continuous tissue-optical monitoring (adapted with permission from ref 43, 10.1088/1361-6579/acead2). An underside view highlights the emitter–detector layout and optical window for skin interfacing.

**Figure 5:**
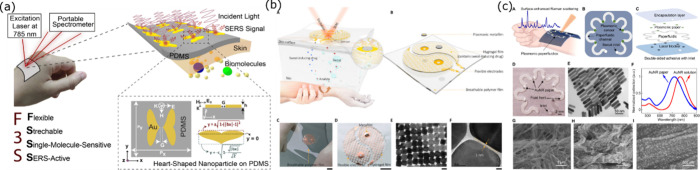
(a) Concept illustration of the proposed plasmonic SERS sensor that can be laminated on the wrist and used with a portable Raman spectrometer for label-free biochemical (sweat) analysis in real time [b]. (b) Wearable SERS device with plasmonic metamaterial. Schematic of the device’s working principle and design [e]. (c) Conceptual illustration of a wearable plasmonic paper-fluidic device for sweat collection, storage, and in situ analysis using SERS [f].

Optical fibers offer another platform for wearable photonic sensor technology with high sensitivity to temperature, pressure, strain, and other physical signals.^63^ Various types of optical fibers such as hydrogel,^64^ PDMS,^65^ and silica microfibers embedded in PDMS^66^ have been applied to wearable sensor platforms and can provide mechanical flexibility and biocompatibility. However, existing optical fiber-based technologies have limitations in terms of integration level, large-scale manufacturing processes, and spatial design freedom.^67^ To overcome these challenges, a flexible integrated photonics technology has been proposed for wearable sensing. Flexible integrated photonic architectures have recently enabled wearable optical sensing platforms for motion and physiological monitoring (Figure 6). They can integrate multifunctional units on the micrometer scale while maintaining the high sensitivity and immunity to electromagnetic interference of optical fiber sensors.^68^ Recently, various next-generation platforms have been reported, such as flexible silicon photonics, single-mode stretchable optical waveguides, and bendable and stretchable integrated photonic sensors.^69,70^ Nevertheless, fully realized prototypes for direct skin-attachment wearable systems are limited to date, and further research is required to address long-term wear stability, mechanical durability, biocompatibility, and integration of real-time data processing. Looking ahead, the field of wearable sensors is moving toward convergent platforms that deliberately pair photonic channels with electrochemical readouts, using orthogonal sensing to improve specificity, correct drift, and maintain accuracy under real-world perturbations. The most scalable path will couple such multimodal hardware with closed-loop calibration and on-device inference that explicitly accounts for motion, environment, and interindividual variability.^71^ The quantitative reliability for wearable photonic biosensing is restricted due to substrate-to-substrate SERS variability, optical losses, skin curvature, alignment changes caused by motion, the thickness of the sweat films, the photothermal heating, and background fluorescence. All of these factors contribute to the difficulties of calibration and reproducibility compared with benchtop optical assays and to the necessity for integrated reference structures, ratiometric analysis, and co-registered electrochemical validation.

**Figure 6:**
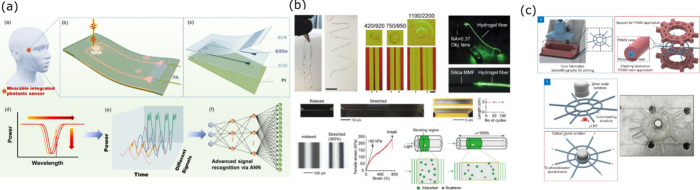
(a) Design architecture and working principle of the wearable integrated photonic sensor for monitoring human motion and physiological signals. The wearable integrated photonic sensor for motion and physiological monitoring with a packaged wearable integrated photonic sensor within multilayers of different materials [d]. (b) Optical and mechanical properties of step-index, core/clad alginate-polyacrylamide hydrogel fibers [e]. (c) Schematic representation of the core/cladding fabrication steps. Representation of the batteries and μLED assembly in the structure, which is used as the light source of the optical system and proof mass of the structural design [f].

## 4 Electrochemical and Photonic Sensing Complementarity

Electrochemical and photonic biosensing modalities probe different physical dimensions of the identical nano–bio interface and thus are very sensitive to biochemical variations, but they are also vulnerable to perturbation caused by fouling, electrode polarization, and local chemical variations.^72^ By contrast, photonic transduction can detect optical property changes, e.g., refractive index, absorption, scattering, and plasmonic field enhancement, that are a result of molecular adsorption, interfacial restructuring, and dielectric modulation at the same interface. Optical signals therefore highlight structural and physicochemical features of the nano–bio interface, such as adsorption kinetics, conformational transitions, and surface morphology, which do not necessarily lead to immediate electrochemical signals. Notably, electrical and optical observables cannot be considered independent but rather interact with each other due to common interfacial processes, i.e., molecular accumulation, hydration state, and nanoscale structural evolution. Complementarity between the electrochemical and photonic sensing lies between their abilities to probe similar and yet nonidentical sets of the interfacial state space.^73^ Whereas electrochemical measurements can access biochemical reactivity and charge dynamics directly, photonic measurements are able to find a nonperturbative window into interfacial organization and material biomolecule interactions. Their cross allows discerning of their interfaces, which makes them more observable in young biochemical changes, artifacts due to mechanical deformations, or other environmental fluctuations or material degradation. In wearable systems, where physiological conditions keep changing, the dual point of view is crucial to obtaining strong and interpretable sensing when the system is deployed in the real world.

## 5 Convergent Electrochemical Photonic Architectures: Design Models

New applications of hybrid electrical and optical sensors in microsystems have offered interesting design paradigms for convergent wearable biosensing systems. Electrochemical transduction and optical interrogation lab-on-a-chip systems^74^ have shown that spatially localized electrochemical assays and biomolecular interaction assays can coexist in the same cell without interference with their spatial localization or functional specificity. These systems confirm the co-fabrication, sensing, and co-interpretation of electrochemical and photonic elements in one device. As per the design, this convergence allows the intentional overlap of electrical and optical volumes of sensing to make sure that both modalities probe the same nano–bio interface as opposed to spatially and temporally independent regions. It is necessary that this colocalization exists to make multimodal data fusion meaningful because it allows electrical and optical data to be viewed as correlated effects of the same interfacial processes. More importantly, these architectures prove that multimodal integration is not necessarily associated with trade-offs in miniaturization, power efficiency, or mechanical compliance. The upscaling of these convergent architectures of benchtop microsystems into wearable systems will attest to the fact that the integration of electrochemical–photonic systems is not a conceptual abstraction. Flexible substrates, textile-integrated electrodes, and skin-mounted platforms can accommodate both the electrical and optical components. These models offer a structural base for converting convergence into wearable diagnostics, where multimodal sensing will be integrated into dressings, garments, or epidermal patches and will not interfere with the functionality or the comfort of the user.

## 6 Data-Driven and AI-Enabled Multimodal Wearable Biosensing

To make convergent electrochemical–photonic wearables from platforms into clinically reliable diagnostic systems, data-driven analysis is increasingly demanded. In wearable operation, signal quality is continuously perturbed by motion artifacts, biofouling, hydration variability, mechanical deformation, optical attenuation, and interfacial drift, making static calibration approaches insufficient for long-term monitoring. Recent research in AI-enabled wearable biosensing emphasize the challenge of gathering signals, which is not the only issue; rather, the most pressing need is strong interpretation of complex multimodal biosignals in dynamic physiological conditions.^75^ Machine learning and deep-learning frameworks have been increasingly adopted to compensate for baseline drift, compensate for drift, perform adaptive filtering, detect anomalies, and reject motion artifacts and for personalized calibration of wearable biosensors. In addition, AI-assisted biosensing studies further reveal the potential of CNN, RNN, transformer, and ensemble-learning models to enhance signal extraction, mitigate environmental interference, and enable multimodal fusion in real time in the low signal-to-noise regime.^76^ AI is especially significant for convergent electrochemical–photonic systems where electrical and optical signals are two nonredundant observables of the same nano–bio interface which are mechanistically coupled. Electrochemical channels can be used for probing electron-transfer kinetics, ionic transport, and interfacial impedance, while photonic channels can be used for probing the refractive index modulation, scattering, plasmonic enhancement, and adsorption dynamics. The recent fusion of biochemical, electrophysiological, mechanical, and imaging data across multiple domains using AI frameworks has shown that the fused data yield more robust diagnostics and more physiological-context interpretation than data from these individual sensing modalities alone.^77^ Importantly, these frameworks are increasingly adopting temporal synchronization, fusion based on causality, adaptive calibration, and learning over time, concepts that are directly applicable to electrochemical–photonic wearable systems. Therefore, the assessment of converging electrochemical–photonic wearable systems has to move beyond the classic limits of detection and sensitivities and consider mechanistically and operationally relevant figures-of-merit, such as the stability of the interface, the ability to synchronize multiple modalities, the robustness of the wearable architecture, and the interpretability of the signals across modalities in dynamic physiological environments (Table 2).

**Table 2:** Mechanistically Relevant Figures-of-Merit for Convergent Electrochemical–Photonic Wearable Biosensing Systems

Figure-of-merit	Electrochemical relevance	Photonic relevance	Importance in convergent wearable systems
**Interfacial drift stability**	Electrode fouling, reference instability, redox drift, impedance variation	Optical baseline drift, scattering fluctuations, plasmonic instability	Determines long-term reliability and calibration fidelity during continuous wearable operation
**Cross-modal synchronization fidelity**	Temporal alignment of electrochemical response kinetics	Temporal consistency of optical acquisition and spectral response	Ensures both modalities interrogate the same physiological and biochemical event
**Fouling resistance**	Protein adsorption, biofilm formation, loss of electroactive area	Optical attenuation, surface contamination, refractive-index perturbation	Critical for maintaining signal reproducibility at dynamic nano–bio interfaces
**Mechanical-operational stability**	Signal retention under strain, bending, and micromotion	Optical coupling stability during deformation and motion	Governs real-world wearable robustness under ambulatory conditions
**Multimodal interpretability**	Correlation of current, impedance, and electrochemical dynamics	Correlation of spectral, scattering, and plasmonic responses	Enables AI-assisted discrimination between true biochemical events and environmental artifacts

## 7 Toward Adaptive, Information-Rich, and Clinically Viable Wearable Diagnostics

The integration of electrochemical and photonic biosensing is a fundamental step toward adaptive wearable diagnostics, which are able to sample multidimensional physiological information in continuously changing and dynamic nano–bio interfaces. Through co-designing electrical and optical transduction modalities, wearable platforms have the potential to query biochemical activity, interfacial structure, and microenvironmental conditions at the same time and address the ambiguity and drift inherent in single-modal sensing. Such convergence not only adds information, but it also allows cross-validation across modalities, and this is necessary in maintaining measurement fidelity in all aspects of wearable devices such as mechanical deformation, changes in hydration, and biochemical fouling that are inherent to real-world wear (Figure 7). Moreover, as wearable systems change from being more of a short-term demonstration to a long-term deployment, bioacceptability, material safety, and regulatory compliance become central design constraints and not additional considerations. Long-term contact of the sensor to the skin potentially exposes the user to conductive inks, nanostructured electrodes, encapsulants, and interconnect materials. Here, convergent electrochemical–photonic structures for convergent bioimpediments represent an untapped exploitation of surveying not only physiological biomarkers but also device–tissue communications per se. In principle, optical modalities that are sensitive to interfacial morphology, dielectric changes, or scattering may be co-opted to monitor encapsulation integrity, fouling dynamics, or inflammatory microenvironments, and electrochemical channels can still report biochemical activity. These considerations are closely related, translationally, to new regulatory requirements emerging in wearable and skin-interfaced medical devices, which are more focused on long-term safety, material characterization, and consistency of postmarket performance. In the future, wearable diagnostic devices that will have the most focus will be those that view convergence not as multimodal redundancy but as a systems-level approach that brings together sensing, validation, and safety through a common nano–bio interface. With electrochemical sensitivity to biochemical flux and photonic sensitivity to interfacial structure and material state, future wearable platforms can scale to systems that are not only portable and continuous but also information rich, adaptive, and biologically responsible and can perform reliably across the broad range of users and across extended time scales.

**Figure 7:**
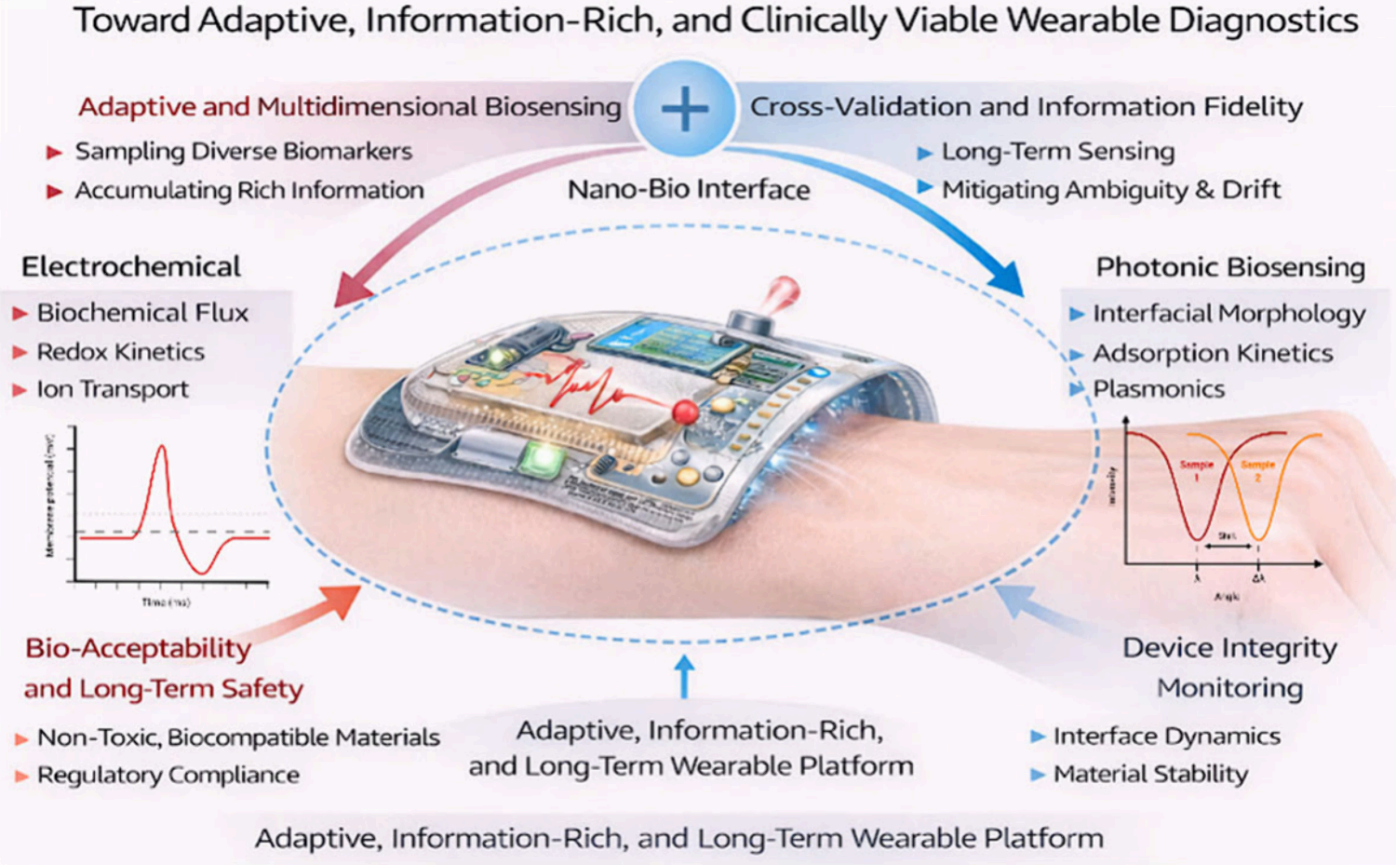
Toward adaptive, information-rich, and clinically viable wearable diagnostics.
